# Prescription Analysis in the Digital Era: Comparing Artificial Intelligence-Based Versus Manual Approaches

**DOI:** 10.7759/cureus.114980

**Published:** 2026-08-22

**Authors:** Gaurav Kakasaniya, Ruchita J Mer, Dimple S Mehta, Sunita Chhaiya, Tejas Acharya, Madhav Trivedi, Mauli Sanghvi, Anand Zatiya, Jimika Ved, Maitri Patel

**Affiliations:** 1 Department of Pharmacology, C. U. Shah Medical College and Shrimad Rajchandra Sarvamangal Hospital, Surendranagar, IND

**Keywords:** artificial intelligence, essential medicines list, manual analysis, prescription analysis, who prescribing indicators

## Abstract

Background

Prescription auditing using the World Health Organization (WHO) core prescribing indicators is a standard method to evaluate rational drug use. With advances in artificial intelligence (AI), AI-based tools may offer faster approaches for prescription analysis; however, comparative evidence with manual analysis remains limited. The objective of this study was to compare AI-based prescription analysis with manual prescription analysis using WHO core prescribing indicators.

Methodology

An observational study was conducted at a tertiary care teaching hospital over a period of three months. A total of 308 outpatient prescriptions were collected from the Medicine, Obstetrics and Gynecology, and Orthopedics outpatient departments. Prescriptions were analyzed manually according to WHO core prescribing indicators. AI-based analysis was performed using Google Gemini Pro (Google LLC, Mountain View, California). Results from both approaches were compared using a paired t-test and the chi-square test.

Results

AI-based prescription analysis produced results largely comparable to manual analysis for most WHO prescribing indicators. No significant differences were observed between AI and manual analysis for most prescribing indicators. However, a significant difference was noted in the percentage of medicines prescribed from the essential medicines list in the Medicine and Obstetrics and Gynecology departments.

Conclusion

AI-based prescription analysis demonstrated results broadly comparable to conventional manual analysis for most WHO core prescribing indicators. These findings suggest that AI-assisted tools may serve as a feasible supportive approach for prescription auditing.

## Introduction

Rational use of medicines is a cornerstone of effective healthcare delivery, ensuring that patients receive medications appropriate to their clinical needs, in correct doses, for an adequate duration, and at the lowest possible cost to both the patient and the community. The concept of rational drug use has been strongly emphasized by the World Health Organization (WHO) as an essential strategy for improving therapeutic outcomes and reducing unnecessary healthcare expenditure [1].

To monitor prescribing practices and promote rational drug use, the WHO, in collaboration with the International Network for Rational Use of Drugs (INRUD), developed standardized core drug-use indicators. These indicators include parameters such as the average number of medicines per encounter, the percentage of medicines prescribed by generic name, the percentage of encounters with an antibiotic prescribed, the percentage of encounters with an injection prescribed, and the percentage of medicines prescribed from the essential medicines list (EML) [2]. These indicators are widely used in healthcare facilities to assess prescription patterns and identify irrational prescribing practices such as polypharmacy, excessive antibiotic use, or deviation from essential medicine guidelines [3].

Manual prescription analysis based on WHO core prescribing indicators has been extensively conducted in both inpatient and outpatient settings to evaluate drug utilization patterns and prescribing behavior. Such studies provide important insights into prescribing trends and help in improving rational drug use through prescription auditing and feedback to prescribers [3,4]. Regular prescription audits are therefore considered an important strategy for identifying inappropriate prescribing practices and promoting rational pharmacotherapy [4].

In recent years, the rapid advancement of digital technologies and artificial intelligence (AI) in healthcare has opened new opportunities for data analysis and clinical decision support. AI-based systems are capable of processing large volumes of healthcare data rapidly and systematically. Their application in prescription analysis could enhance efficiency, reduce manual workload, and enable continuous monitoring of drug utilization patterns. However, despite these potential advantages, it is essential to evaluate whether AI-based analyses yield results comparable to those of traditional manual prescription auditing, which remains the established and widely accepted method for assessing prescribing indicators [5,6].

In tertiary care teaching hospitals, where prescription patterns reflect diverse clinical conditions and real-world therapeutic practices, evaluating such methods becomes particularly relevant. Therefore, the objective of the present study was to compare manual prescription analysis with AI-based prescription analysis using WHO core prescribing indicators.

## Materials and methods

Study design and setting

This was an observational data analysis study conducted at a tertiary care teaching hospital in Surendranagar, Gujarat, India.

Ethical approval

Approval for the study was obtained from the Institutional Ethics Committee (approval number: CUSMC/IEC(HR)/RP-4/2026/Final Approval/41/2026) of the tertiary care teaching hospital before the initiation of the study.

Study duration

The study included prescription data collected over a three-month period from July 2025 to September 2025.

Study sample and data collection

A total of 308 outpatient prescriptions were collected from the Medicine, Obstetrics and Gynecology (OBGY), and Orthopedics outpatient departments during the study period. The department-wise distribution of prescriptions was as follows: Medicine, 130 prescriptions; Orthopedics, 96 prescriptions; and OBGY, 82 prescriptions.

Only prescriptions containing complete drug details and legible drug information were included in the study. During the study period, a total of 320 prescriptions were screened. Twelve prescriptions were excluded because of incomplete or illegible drug information, and the remaining 308 prescriptions were included in the final analysis.

Manual prescription analysis

Manual prescription analysis was performed using the WHO core prescribing indicators. The manual prescription analysis was initially performed by resident doctors, who assessed the prescriptions according to the WHO core prescribing indicators. The data were subsequently reviewed by a faculty member of the department as a second reviewer to ensure the accuracy and consistency of the analysis. Any discrepancies identified during the review were discussed and resolved before finalizing the data. The following indicators were calculated [7]: average number of drugs per encounter, percentage of drugs prescribed by generic name, percentage of encounters with an antibiotic prescribed, percentage of encounters with an injection prescribed, and percentage of drugs prescribed from the EML.

Drug-related data from each prescription were first entered into a Microsoft Excel spreadsheet (Microsoft Corporation, Redmond, Washington), following which manual prescription analysis was performed and the above WHO core prescribing indicators were calculated according to the standard WHO methodology.

AI-based prescription analysis

For AI-based analysis, Google Gemini 3.1 Pro (Google LLC, Mountain View, California) was used. Drug details from each prescription were compiled into a Microsoft Word file (Microsoft Corporation, Redmond, Washington), which was then entered into the AI system. The AI input included the drug name, strength, dose, dosage form, and route of administration as documented in each prescription. Thus, the AI analysis was performed using the relevant drug-related information available in the original prescriptions.

The AI model was instructed to analyze outpatient prescriptions using the WHO core prescribing indicators and the National List of Essential Medicines (NLEM) 2022. The standardized prompt instructed the model to assess the average number of medicines per encounter, generic prescribing, antibiotic use, injection use, and the proportion of medicines prescribed from the NLEM 2022. The model was instructed to use the provided drug name, strength, dose, dosage form, and route of administration for the analysis and to present the results in a structured format.

The AI-generated results included the same prescribing indicators evaluated during manual analysis: average number of drugs per encounter, percentage of drugs prescribed by generic name, percentage of encounters with an antibiotic prescribed, percentage of encounters with an injection prescribed, and percentage of drugs prescribed from the NLEM 2022.

To ensure data privacy, no patient identifiers were entered into the AI system. Only drug-related information required for prescription analysis was used. Thus, the confidentiality and anonymity of patient data were maintained throughout the study.

Statistical analysis

Data from both manual and AI-based prescription analyses were compared using appropriate statistical methods. The average number of drugs per encounter was compared using an independent two-tailed t-test. Categorical prescribing indicators, including the percentage of drugs prescribed by generic name, percentage of encounters with an antibiotic prescribed, percentage of encounters with an injection prescribed, and percentage of drugs prescribed from the EML, were analyzed using the chi-square test. Statistical analysis was performed using Epi Info™ version 7.2 software (Centers for Disease Control and Prevention, Atlanta, Georgia). A p-value of <0.05 was considered statistically significant.

## Results

A total of 308 outpatient prescriptions were included in the study from three departments of the tertiary care teaching hospital: Medicine (n = 130), Orthopedics (n = 96), and OBGY (n = 82). Prescription analysis was performed using both manual evaluation and AI-based analysis using Google Gemini Pro according to the WHO core prescribing indicators. Comparisons between the two methods were evaluated using the independent t-test and chi-square test.

Medicine department

The comparison of WHO core prescribing indicators between manual and AI-based prescription analysis in the Medicine department is shown in Table 1.

**Table 1:** Comparison of AI-based and manual prescription analysis using WHO core prescribing indicators in the Medicine department Data are presented as the mean for the average number of drugs per encounter and percentages (%) for categorical variables. Statistical significance was considered at p < 0.05. *Statistically significant. A t-test was used to compare the average number of drugs per encounter, while the chi-square (χ²) test was applied to categorical prescribing indicators. EML: essential medicines list.

Indicator	AI	Manual	Statistical analysis	P value
Average number of drugs per encounter	4.12	4.37	t = 1.9564	0.052
Percentage of drugs prescribed by generic name	1.31%	2.24%	χ² = 1.3586	0.2438
Percentage of encounters with an antibiotic	9.23%	1.87%	χ² = 0.1986	0.6559
Percentage of encounters with an injection	6.15%	2.05%	χ² = 0.0000	1.000
Percentage of drugs from EML	23.32%	39.32%	χ² = 31.8614	<0.001*

In the Medicine department, the average number of drugs per encounter was slightly higher in manual analysis than in AI analysis; however, the difference was not statistically significant. Similarly, the percentage of drugs prescribed by generic name, percentage of encounters with an antibiotic prescribed, and percentage of encounters with an injection prescribed did not show significant differences between the two approaches. However, the percentage of drugs prescribed from the EML (National List of Essential Medicines 2022) was significantly higher in manual analysis than in AI-based analysis (p < 0.001).

OBGY department

The comparison of prescribing indicators for the OBGY department is presented in Table 2.

**Table 2:** Comparison of AI-based and manual prescription analysis using WHO core prescribing indicators in the Obstetrics and Gynecology department Data are presented as the mean for the average number of drugs per encounter and percentages (%) for categorical variables. Statistical significance was considered at p < 0.05. *Statistically significant. A t-test was used to compare the average number of drugs per encounter, while the chi-square (χ²) test was applied to categorical prescribing indicators. EML: essential medicines list.

Indicator	AI	Manual	Statistical analysis	P value
Average number of drugs per encounter	2.27	2.25	t value = - 0.2207	0.825
Percentage of drugs prescribed by generic name	8.60%	9.72%	χ² = 0.1417	0.7066
Percentage of encounters with an antibiotic	17.07%	9.18%	χ² = 0.2100	0.6468
Percentage of encounters with an injection	4.88%	27.77%	χ² = 0.1492	0.6993
Percentage of drugs from EML	24.73%	48.10%	χ² = 21.8971	<0.001*

In the OBGY department, the average number of drugs per encounter was similar between manual and AI-based analyses. The percentage of drugs prescribed by generic name, percentage of encounters with an antibiotic prescribed, and percentage of encounters with an injection prescribed were also comparable between the two methods and did not show statistically significant differences. However, the percentage of drugs prescribed from the EML was significantly higher in manual analysis than in AI analysis (p < 0.001).

Orthopedics department

The comparison of WHO prescribing indicators between manual and AI-based prescription analysis in the Orthopedics department is presented in Table 3.

**Table 3:** Comparison of AI-based and manual prescription analysis using WHO core prescribing indicators in the Orthopedic department Data are presented as the mean for the average number of drugs per encounter and percentages (%) for categorical variables. Statistical significance was considered at p < 0.05. A t-test was used to compare the average number of drugs per encounter, while the chi-square (χ²) test was applied to categorical prescribing indicators. EML: essential medicines list.

Indicator	AI	Manual	Statistical analysis	P value
Average number of drugs per encounter	2.20	2.21	t = - 1.1409	0.256
Percentage of drugs prescribed by generic name	0.95%	2.34%	χ² = 1.2787	0.2581
Percentage of encounters with an antibiotic	0.1%	0.46%	χ² = 1.0052	0.3161
Percentage of encounters with an injection	0%	0%	χ² = 0.0000	1.000
Percentage of drugs from EML	25.12%	22.53%	χ² = 0.3898	0.5324

In the Orthopedics department, the values obtained from manual and AI-based prescription analyses were largely similar across all WHO core prescribing indicators. No statistically significant differences were observed between the two approaches for the average number of drugs per encounter, generic prescribing, antibiotic prescribing, injection prescribing, or percentage of drugs from the EML.

Overall, AI-based prescription analysis showed findings broadly similar to those of manual analysis for several WHO core prescribing indicators across the three departments. However, differences were observed for certain indicators, particularly the percentage of drugs prescribed from the EML in the Medicine and OBGY departments.

## Discussion

The present study compared AI-based prescription analysis with traditional manual prescription auditing using WHO core prescribing indicators across three clinical departments. The results demonstrate that AI-assisted analysis yielded findings broadly comparable to manual evaluation for most prescribing indicators, although certain discrepancies were observed in specific parameters, such as antibiotic encounters and the identification of drugs from the EML.

In the present study, the average number of drugs per encounter, calculated using AI-based and manual prescription analyses, showed minimal variation across the Medicine, OBGY, and Orthopedics departments, and statistical comparison did not reveal a significant difference between the two approaches. This suggests that AI systems can reliably quantify prescription-related data similarly to conventional human analysis. Similar observations have been reported in recent studies evaluating artificial intelligence in healthcare data analysis, in which AI systems demonstrated performance comparable to human evaluators across various clinical assessment tasks [8].

The percentage of drugs prescribed by generic name showed small differences between AI and manual analyses in the present study. Although the values were slightly lower in AI-based evaluation, the difference was not statistically significant across departments. This suggests that AI-based tools are capable of identifying prescribing patterns related to generic drug use with accuracy comparable to manual review. Similar observations have been reported in studies evaluating AI-supported clinical decision systems, where AI recommendations were largely consistent with clinician prescribing patterns but showed some variability depending on data input and contextual factors [9].

Some variation between AI-based and manual analysis was observed in the percentage of encounters with antibiotics across certain departments, although the difference was not statistically significant. AI-based analysis tended to identify a higher proportion of antibiotic encounters compared with manual analysis in certain settings. One possible explanation is that AI systems rely strictly on algorithm-based recognition of drug categories, whereas manual reviewers may interpret prescriptions differently or overlook certain drug classifications. Previous research on AI-driven prescribing systems has also shown that AI tools can more consistently detect antibiotic prescribing patterns and improve antimicrobial stewardship by identifying inappropriate antibiotic use [10].

Similarly, some variation was observed in the percentage of encounters with injections, particularly in the OBGY department, where manual analysis identified a higher proportion of injection use compared with AI-based analysis. This may reflect differences in the classification or identification of injectable drugs between AI-generated analyses and manual evaluations. Human reviewers may use contextual knowledge while categorizing drugs, whereas AI systems rely strictly on algorithm-based classification of the provided drug information.

A statistically significant difference between AI and manual analyses was observed in the percentage of drugs prescribed from the EML across some departments. Manual analysis identified a higher proportion of drugs from the EML compared with AI analysis. This difference may be explained by limitations in the drug database used by the AI system or difficulty in mapping brand names to their corresponding generic names listed in the national EML. Similar limitations of AI-based healthcare analytics have been reported in recent literature, where the accuracy of AI outputs was found to depend strongly on the quality, completeness, and standardization of input data and reference databases [11].

Overall, the findings of the present study suggest that AI-based prescription analysis produces results broadly comparable to manual prescription auditing for most WHO prescribing indicators. However, certain discrepancies were observed in parameters such as essential drug identification and antibiotic encounters, indicating that AI tools may require further refinement in drug classification and database integration.

Strengths of the study

The present study has several strengths. It directly compares AI-based prescription analysis with conventional manual analysis using standardized WHO core prescribing indicators, enabling objective assessment of agreement between the two methods. Prescriptions from multiple departments, Medicine, OBGY, and Orthopedics, were included, reflecting diverse real-world prescribing practices in a tertiary care teaching hospital. The use of a defined sample size and appropriate statistical tests (t-test and chi-square test) strengthens the reliability of the comparison. In addition, the study demonstrates the practical use of an AI tool (Google Gemini Pro) for prescription auditing. Importantly, as only limited studies have compared AI-based and manual prescription analysis using WHO indicators, this study provides novel evidence supporting the feasibility of AI-assisted prescription auditing for monitoring rational drug use.

Limitations of the study

This study has certain limitations. First, it was conducted at a single tertiary care teaching hospital, which may limit the generalizability of the findings to other healthcare settings. Second, the analysis was performed using a single AI platform, Google Gemini 3.1 Pro; therefore, the findings may not necessarily be applicable to other AI-based tools. In addition, AI-generated outputs may vary depending on the model version, input structure, and prompting approach, which may affect the reproducibility of the findings. Finally, the study was based on prescription data collected during a specific three-month study period, which may limit the broader applicability of the findings.

## Conclusions

The present study compared AI-based prescription analysis with conventional manual analysis using WHO core prescribing indicators in a tertiary care teaching hospital. The findings showed that AI-generated results were comparable with manual analysis for most prescribing indicators, with only minor variations observed in certain parameters. These results suggest that AI-based prescription analysis may serve as a supportive tool for prescription auditing and monitoring of prescribing practices. Further validation using larger datasets, multiple AI platforms, and independent agreement assessment is required before AI-based analysis can be considered a substitute for conventional manual review.
